# Experiences and Management of Distress and the Use of Music, Including Music Therapy, on NHS Inpatient Mental Health Dementia Wards: A Qualitative Study

**DOI:** 10.1002/gps.70091

**Published:** 2025-05-02

**Authors:** Naomi Thompson, Rachel Hunt, Helen Odell‐Miller, Abdulwarrith Olawale, Lucy Pickering, Chris Pointon, Benjamin R. Underwood, Alison Wilkinson, Christine Wise, Emma Wolverson, Ming‐Hung Hsu

**Affiliations:** ^1^ Cambridge Institute for Music Therapy Research Anglia Ruskin University Cambridge UK; ^2^ Arts Therapies Services Cambridgeshire and Peterborough NHS Foundation Trust Fulbourn Hospital Fulbourn UK; ^3^ Public Contributor Cambridge Institute for Music Therapy Research Anglia Ruskin University Cambridge UK; ^4^ Cambridgeshire and Peterborough NHS Foundation Trust Fulbourn Hospital Fulbourn UK; ^5^ East London NHS Foundation Trust London UK; ^6^ Inpatient Dementia Patient and Public Involvement Group University of Hull Hull UK; ^7^ Department of Psychiatry University of Cambridge Cambridge UK; ^8^ Faculty of Science and Engineering Anglia Ruskin University Cambridge UK; ^9^ Dementia UK London UK; ^10^ Faculty of Health Sciences University of Hull Hull UK

**Keywords:** co‐design, dementia, distress, inpatient mental health dementia wards, music, music therapy, qualitative study

## Abstract

**Background:**

Inpatient mental health dementia wards in the National Health Service (NHS) provide specialist care for people with dementia experiencing acute levels of distress. There is little research exploring how best to manage and prevent distress. Music therapy may be feasible to deliver and reduce the prevalence of distress behaviours.

**Aims:**

To further understanding of experiences of distress in inpatient mental health dementia NHS wards, how distress is managed and ways music and music therapy is used.

**Methods:**

Semi‐structured focus groups and interviews were co‐designed and conducted with people with dementia, families, staff, music therapists and managers with experience of this setting. Data were transcribed and analysed using reflexive thematic analysis, with findings corroborated with participants, a co‐design group and experts‐by‐experience.

**Results:**

49 people took part from 17 wards. Three overarching themes were identified, with 10 subthemes. The first theme highlighted the complex physical and mental health care needs of people on these wards, including high levels of distress. Secondly, staff and families aimed to personalise care to manage and prevent distress. Thirdly, music, including music therapy, could support the delivery of personalised care and help prevent and deescalate distress behaviours, potentially reducing the need for restrictive interventions. However, managers, staff, families and patients reported that care provision did not always meet patient need and resource limitations often prevented delivery of personalised care.

**Conclusions:**

NHS mental health dementia wards provide specialist care for people with dementia experiencing high levels of distress. Personalised care, including the use of music, was essential for preventing and managing distress, and could be enhanced through specialist support from a music therapist. Findings should inform best practice guidelines for NHS inpatient mental health dementia wards, including the use of music and music therapy, to support the prevention and management of distress for this client group.

**Summary**

- Inpatient mental health dementia wards care for people experiencing crisis often resulting in complex and acute distress. But little is known about how best to care for this population.
- Staff aim to understand causes of distress through communicating with families and multidisciplinary healthcare professionals so that they can personalise care and treatment.
- Patient and family experiences of inpatient mental health dementia care in the NHS vary. It is not always possible to identify causes and reduce distress owing to the complexity of presentation and inadequate funding for resources and staffing.
- Using music is an accessible, non‐verbal way to personalise care, helping to prevent and manage distress. A music therapist can provide specialist treatment and assess how music can be formalised as part of an individual's care.

## 1 Introduction

Inpatient mental health dementia wards, also known as psychiatric wards, provide care for people with dementia requiring specialist support. These individuals experience acute levels of distress which is putting their safety or the safety of others at risk [1, 2]. The term distress refers to behavioural changes often experienced by people with dementia. Presentations may include agitated behaviours such as shouting, throwing and kicking or non‐agitated behaviours such as pacing with purpose, crying, withdrawal and resistance to care/medication [3]. The language used has been chosen to align with preferred language by people with dementia reflecting that distress can be caused by symptoms of dementia and/or be an expression of unmet needs [4, 5, 6, 7]. In England and Wales, people with dementia may be detained on National Health Service (NHS) wards under the provisions of the Mental Health Act 2007 or Deprivation of Liberty Safeguards, meaning they can be treated without their consent [8]. Wards aim to assess and treat the crisis surrounding the person with dementia [9, 10]. Admission often follows a breakdown of care in the home or care home which can be traumatic for the person with dementia and their family member(s) [8, 11]. Caring for this population is complex as many have multiple long term conditions and may be nearing the end of life, yet there is a lack of research and heterogeneity in the delivery of care [2, 12].

There are still significant gaps in dementia care research and practice but evidence‐based recommendations for care in the UK state that psychosocial interventions should be the first line of treatment for distress behaviours in dementia care [13, 14]. Research also shows that pharmacological interventions, such as antipsychotic medication, are frequently used with limited benefit and concomitant increase in adverse effects including associations with physical illness and death [1, 15, 16, 17]. A recent systematic review found limited research with varying methodological quality into psychosocial interventions on inpatient mental health dementia wards internationally [18]. However, there were some indications that these interventions, in particular music therapy^1^ [13], and multisensory interventions, may reduce experiences of distress when delivered by trained interventionists in a person‐centred, accessible way. Additionally, a realist review looked at how and why music therapy reduces distress and improves wellbeing for people with advanced dementia in institutional settings [19]. The programme theory highlighted the need for music therapists to assess and meet unmet needs in the moment to facilitate short term reductions in distress. The trained therapist should then work collaboratively with staff and families to embed personalised music interventions in the daily prevention and management of distress. Despite the potential helpfulness of music therapy, an audit of practice on these wards in the UK in 2020 reported that access to this intervention is limited with a wide range in contracted hours limiting the therapists' ability to deliver individualised interventions [20].

While emerging evidence and programme theory suggest that music therapy may be feasible and helpful to reduce distress on inpatient mental health dementia wards, there is little understanding of how distress is experienced and whether and how music and music therapy are currently used in practice on NHS wards. This qualitative study sought to further understanding of how managers, staff, people with dementia and families experience distress in NHS inpatient mental health dementia wards. This included how distress was managed, and ways in which music was or was not used in this process. The research was undertaken as part of the MELODIC (Music therapy Embedded in the Life Of Dementia Inpatient Care) study (NIHR204928) [21]. This is a co‐design project, with the academic team working alongside a team of people with personal and professional experience of similar wards at all stages of research design, data collection, analysis and interpretation [22, 23]. The authors assert that all procedures contributing to this work comply with the ethical standards of the relevant national and institutional committees on human experimentation and with the Helsinki Declaration of 1975, as revised in 2013. All procedures involving human participants were approved by the Health Research Authority (IRAS, no. 323503), and Anglia Ruskin University (ETH2223‐8044). The research questions, reached following collaborative co‐design workshops, were:

1. How do people with dementia, families, staff and managers experience distress on inpatient mental health dementia wards?
2. How is distress currently managed?
3. How is music currently used by people with dementia, families, staff and managers on wards?
4. Does the use of music impact on experiences of distress?

## 2 Methods

A qualitative study was undertaken from November 2023–April 2024 using semi‐structured focus groups and interviews and reflexive thematic analysis [24, 25]. Findings are presented adhering to the COREQ reporting guidelines [26].

### 2.1 Participants

Participants were recruited from the following groups: people with dementia, carers and families, staff working on wards, music therapists and ward managers. A heterogenous sample was sought to increase understanding of the experiences and priorities of all involved with mental health dementia wards, enabling future research and policy to seek changes in practice that are meaningful, acceptable and possible to implement. We aimed to recruit 25–36 participants with seven to nine people in each participant group. This target was set to enable the inclusion of diverse experience of mental health dementia wards across multiple NHS Trusts, while remaining achievable within the time and resource limitations of the study. Inclusion criteria were:

- Direct or indirect (through family and close friends) experience of inpatient mental health dementia wards in the last 5 years to capture current experiences.
- The ward was part of the NHS, with private care excluded.
- The ward came under NHS mental health provision, with wards situated within general health hospitals excluded.
- The ward was for people with dementia (sometimes called organic) only, with wards caring for people with other mental health illnesses and dementia together excluded.
- The participant must be able to speak English.
- No geographical restrictions within the UK.

### 2.2 Recruitment

Purposive sampling was used to include people with various experiences, demographics, geographical locations and multidisciplinary team positions. Different roles within the multidisciplinary team, such as nurses, therapists, doctors and ward managers (who usually have a clinical background) were identified alongside the co‐design group to ensure all positions were represented in the sample. The study was advertised through social media, charitable organisations, patient and public involvement forums, the Clinical Research Network (now Research Delivery Network) and the team's personal and professional networks. Participants were initially approached via email.

### 2.3 Data Collection

Written informed consent was gathered via email, post, or in‐person prior to data collection. Consultees provided consent for people who lacked capacity, with an easy read form used on the day to ensure willingness to take part. Focus groups (approximately 90 min) and interviews (approximately 1 hour) were conducted in parallel in‐person on the ward or online by NT and/or MHH.^2^ Staff members were initially invited to attend a focus group to facilitate multidisciplinary discussion and to increase efficiency of the data collection. Where a participant could not attend a focus group, an interview was offered. Ward managers were only invited to join focus groups with other ward managers to limit hierarchy within the group. Interviews were only offered where this was not possible. Family members and people with dementia were only offered interviews due to the potentially sensitive and distressing content of the interview. People with dementia were supported to participate by a trusted person such as a family or staff member. Participants were informed of the aim of the study and of the facilitators' professional occupations. Three participants had a previous professional relationship with the researchers. As the researchers had not worked closely with any of the participants' or known them personally it was deemed this would not impact on the participants' ability to openly express their views.

The topic guide was informed by the findings of two literature reviews, further developed and tested by the co‐design group (Table 1) [18, 19]. The phrasing and order of the questions were adapted by the facilitators based on participant response, exploring areas of interest arising. The co‐design group met after initial data collection to review the questions. No changes were made, but it was agreed to provide a co‐designed definition of music therapy for those without prior experience of the intervention (Supporting Information S1).

**TABLE 1:** Focus group and interview topic guide.

1. How would you describe mental health dementia wards in the NHS? What is the aim of the inpatient stay on the ward where you work/the ward where your relative is staying?
2. What does distress or crisis look like on the ward where you work/the ward where your relative is staying? From your experience, what contributes to distress or crisis?
3. How are distress and crises managed and prevented? Does the care for individuals change during their inpatient stay? What additional support does the ward need?
4. Is music used on the ward? If yes, tell us about when, who by and how music is used. If no, tell us about why music is not used.
5. How would you describe music therapy? Do you think music therapy is different to using music on wards?
6. Does music and/or music therapy have any impact on experiences of distress and crises? What else can music and/or music therapy help with?

Focus groups and interviews were audio‐recorded on two secure devices, then transcribed by NT and a research assistant. Transcripts were sent to participants to check accuracy, with four participants responding, one of whom identified an error for correction.

### 2.4 Data Analysis

A reflexive thematic analysis was conducted by NT using Nvivo, critically appraised by the co‐design team [24, 25, 27]. Following familiarisation, transcripts were coded inductively by NT and scrutinised by EW and MHH to improve trustworthiness and explore potential researcher biases or assumptions. Participant groups were not separated at this stage to build an understanding of shared experiences of the wards. Codes were grouped into subthemes, and subsequently overarching themes. Relationships between participant roles and experience were explored. Emergent themes and subthemes were refined through an iterative process led by NT, EW and MHH alongside the co‐design group to achieve consensus on the final themes and subthemes and support interpretation. Findings were presented and/or sent via email to research participants and members of the Inpatient Dementia Experience Group, facilitated by Dementia UK, to corroborate findings and support interpretation.

## 3 Results

Forty‐nine participants took part in the study (Table 2). There were no refusals to participate and no one dropped out of the study. One person, a family member, was not eligible at screening as their relative had not been admitted to a mental health dementia ward. Participants represented 17 wards within 12 NHS Trusts in England and one in Wales. There were differences in the wards and between NHS Trusts including average length of stay, admission of individuals from the community, mixed or separated by sex, rural and city, admission of functional patients to organic dementia wards and presence of a Community Crisis Team supporting individuals prior to admission to prevent admission where possible. Eight wards currently had a music therapist working part‐time, one had previously had a music therapist, seven had never had a music therapist and for one it was unclear. Fifteen participants in total had experience of music therapy, including seven members of staff, six music therapists and one person with dementia, while one clinical psychologist had experience of music therapy from a care home setting.

**TABLE 2:** Participant demographic data.

Participant role	
Music therapist	6
Physiotherapist	2
Healthcare assistant	4
Activities co‐ordinator	2
Nurse	5
Ward manager	8
Deputy manager	2
Assistant occupational therapist	2
Occupational therapist	2
Psychiatrist	1
Clinical psychologist	2
Assistant psychologist	2
Family member	5
Patient on dementia ward	2
Age (years)	
Mean	48
Range	61
No. female	37
Religion	
Christian	22
None	17
Hindu	2
Atheist	3
Muslim	1
Sikh	1
Pagan	1
Ethnicity	
Black, Black British, Caribbean or African	1
Asian/Asian British	4
White British	39
Mixed or multiple ethnic groups	1
Not stated	2
Time working on the ward (mean)*	58 months
Time spent on the ward (mean)**	6 months
Time since diagnosis (mean)**	37 months

*Note:* Items marked * indicate staff participants only. Items marked ** indicate family and patient participants only.

**FIGURE 1:**
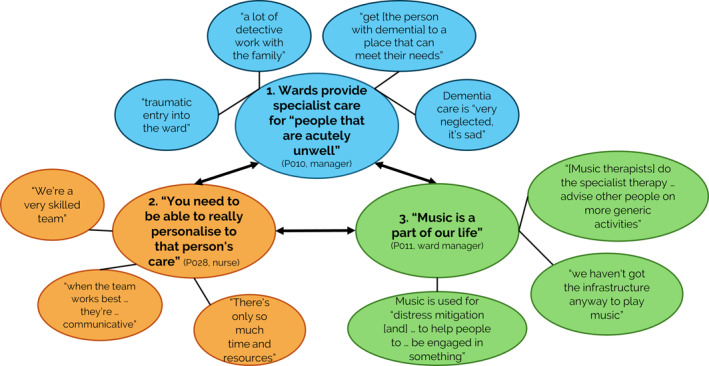
Themes and subthemes derived through reflective thematic analysis.

Three overarching themes with 10 subthemes were identified in relation to experiences and management of distress, and ways that music was used (Figure 1, see Table 3 for additional supporting evidence).

**TABLE 3:** Themes, subthemes derived through reflective thematic analysis with example quotes.

Theme	Subtheme	*Example quote*
Wards provide specialist care for ‘people that are acutely unwell’		‘*People in advanced stages who have quite a severe level of crisis*’ *(P004, music therapist)*
		‘*they're a very poor mix of people at different levels of dementia.*’ *(P024, assistant practitioner)*
		‘*there's a very high level of distress within the wards*’ *(P010, ward manager)*
		‘*it puts a massive strain on the staff as well*’ *(P002, music therapist)*
	‘traumatic entry into the ward’	‘*it's really hard for me in that respect … seeing other people looking after him*’ *(P035, family member)*
		‘*I dread the day he will go back there*’ *(P012, family member)*
	‘a lot of detective work with the family’	‘*It's a very detailed, thoughtful, considered, measured process*’ *(P034, consultant psychiatrist)*
		‘*we've also got to be mindful of … nonverbal ways in which distress can present.*’ *(P034, consultant psychiatrist)*
		‘*I felt fully involved from the beginning*’ *(P037, family member)*
	‘our aim is to get [the person with dementia] to a place that can meet their needs’	‘*hopefully their mental health improves enough that they’re more stable*’ *(P015, nurse)*
		‘*giving plenty of time to the proper processes of preparing a discharge*’ *(P045, ward manager)*
		‘*there’s no care packages so they’re often stuck on the ward*’ *(P004, music therapist)*
	Dementia care is ‘very neglected, it's sad’	‘*I think we don’t know enough about dementia.*’ *(P044, family member)*
		‘*I think historically, you know, funding for older adults isn’t maybe where, at the parity with adults.*’ *(P048, clinical psychologist)*
‘You need to be able to really personalise to that person’s care’		‘*You have to get to know someone on a level where you can … put … ideas in place*’ *(P014, activities coordinator)*
		‘*Being cared for, and to be fair, I think, loved, even*’ *(P037, family member)*
	‘We're a very skilled team’	‘*knowing how to use yourself … as a therapeutic tool, to calm behaviours*’ *(P022, nurse)*
		‘*we've got a really nice environment, we’ve got big garden*’ *(P038, clinical psychologist)*
		‘*We use a lot of sensory activities.*’ *(P026, occupational therapist)*
		‘*without a doubt there is a place for medication*’ *(P026, occupational therapist)*
	‘when the team works best … they're … communicative’	‘*it's important for all staff to genuinely respect each other and the different roles.*’ *(P034, consultant psychiatrist)*
		‘*inviting families in the MDT meetings, discussing the treatment with them and then planning discharge with them*’ *(P010, ward manager)*
		‘*I think it can be quite hierarchical the communication in our ward*’ *(P005, music therapist)*
	‘There’s only so much time and resources’	‘*I do feel that people really want the time and the space and the opportunity to be holistic it’s maybe more of a kind of resource problem.*’ *(P004, music therapist)*
		‘*what’s needed in a ward environment is someone else … to facilitate and plan and organise*’ *(P029, ward manager)*
		‘*we've got a reasonably stocked therapies cupboard*’ *(P045, ward manager)*
‘Music is a part of our life’		‘*music is a language for everybody*’ *(P025, healthcare assistant)*
		‘*So it’s about finding out what someone likes*’ *(P014, activities coordinator)*
		‘*we've got a dedicated day which is Friday, which is kind of music day*’ *(P007, physiotherapist)*
		‘*it's a bit like the blind leading the blind sometimes*’ *(P018, ward manager)*
	Music is used for ‘distress mitigation [and] … to help people to … be engaged in something’	‘*we'll play music … to try and use it as an engagement and activity … to try and prevent escalation.*’ *(P007, physiotherapist)*
		‘*music … can actually deescalate when he’s feeling distressed.*’ *(P038, clinical psychologist)*
		‘*I am a big believer that music brings everybody together*’ *(P013, healthcare assistant)*
	‘we haven’t got the infrastructure anyway to play music’	‘*the place is not built where we can just blast some music for one individual and not impacting others*’ *(P032, nurse)*
		‘*I've noticed that it’s the same few people who use music.*’ *(P007, physiotherapist)*
	‘We [music therapists] do the specialist therapy … advise other people on more generic activities’	‘*I think we hold a much more hopeful stance towards the patients, like it’s very strengths based what we do*’ *(P004, music therapist)*
		‘*So perhaps a music therapist would have … tools in their toolbox that we don’t currently use because we don’t know.*’ *(P026, occupational therapist)*
		‘*Because we’re acutely interacting with someone, we can notice things*’ *(P002, music therapist)*
		‘*I've clawed my way through all of this trying to build up awareness of actually I can do a lot more than just become a human jukebox and try and keep people entertained*’ *(P003, music therapist)*

### 3.1 Theme 1: Wards Provide Specialist Care for ‘People That Are Acutely Unwell’

All participants described the acute and complex mental and physical health needs of people on the ward, who were often in the advanced stages of dementia and sectioned under the provisions of the Mental Health Act 2007. People with differing health needs and mental health diagnoses were often cared for together, complicating care delivery. Distress was frequent, common and extreme, and potential causes were difficult to identify. This impacted on patient, staff and family wellbeing and felt levels of safety. However, staff were reluctant to seek formal support from hospital services provided to support their wellbeing.

#### 3.1.1 Subtheme 1: People With Dementia and Their Families Have a ‘Traumatic Entry Into the Ward’

Admission often followed a progression in the cognitive and/or behavioural presentation of the individual and/or a breakdown in care, which was traumatic for the person with dementia and their family. All participant groups reported that admissions were not always appropriate. In some cases specialist community dementia care was thought to be more appropriate. Admission could cause further deterioration in the person's presentation. Families had complex feelings around admission, including guilt, relief and confusion as to the reason and purpose. Some wards sought to provide support to families, but some families reported not being supported or even recognised by the ward.

9 times out of 10 patients come into [the ward] because the behaviour has changed and it hasn’t been able to be managed within that current environment (P032, nurse)

but to get to that point [admission], why do we have to go through hell? (P044, family member)

#### 3.1.2 Subtheme 2: ‘So a Lot of Detective Work With the Family … so That We Can Create That Care Plan’

The purpose of the admission was to facilitate a detailed, multidisciplinary assessment of the person's distress, which could include diagnosis and provide a holistic multidisciplinary care plan to then discharge the individual in a timely manner to an appropriate placement. However, therapy staff (including physiotherapists, occupational therapists, psychologists and music therapists), ward doctors and one family member reported that triggers for distress were not always identified due to their complexity. This led to frustration and upset for both staff and families and could prolong the inpatient stay. Families were an important part of assessments, sharing their relatives' history and preferences as well as their own caring experiences, but their expertise was not always valued or included.

now [the person with dementia’s] been in a few months now, and his sleep pattern is no better. We’ve had possibly more aggression (P032, nurse)

we also recognise that the carers that are bringing them in have been doing a 24 hour job (P047, ward manager)

#### 3.1.3 Subtheme 3: ‘So Our Aim Is to Get [the Person With Dementia] to a Place That Can Meet Their Needs’

Staff, managers and therapists reported that they aimed to reduce distress so people could be discharged to an appropriate placement and prevent further changes in care. Discharge processes were complex, and location was important to family to enable visiting. However, professional staff reported frequent delays with discharge, mainly due to funding and a lack of specialist care home provision. Prolonged stays on the ward could exacerbate distress for individuals, sometimes meaning that discharge was no longer considered appropriate. However, this may be influenced by the aim of the ward, as some wards were described as providing longer term care for people with dementia experiencing prolonged distress.

once … we’ve reached a degree of stability and we’re not likely to get the patient any better then we start the process of discharge planning. (P034, consultant psychiatrist)

the length of the stay on the wards … all completely depends on the funding. (P009, physiotherapist)

#### 3.1.4 Subtheme 4: Dementia Care Is ‘Very Neglected, It's Sad’

Participants, including managers, therapists and one family member stated that dementia care was neglected in terms of funding, policy and research nationally. This is reflected in the frequent delays with discharge due to a lack of social care funding and appropriate provision as well as the lack of funding and research for inpatient dementia care specifically. Managers' feelings of isolation, lack of support and understanding mirrored family experiences.

it does feel like [whispers] “oh [people with dementia] don’t matter”. It’s not a priority on anybody’s agenda (P017, ward manager)

“I think historically … funding for older adults isn't … at the parity with adults.” (P048, clinical psychologist)

### 3.2 Theme 2: ‘You Need to Be Able to Really Personalise to That Person's Care’

Staff needed to get to know the person with dementia and their families, building a professional loving relationship, to understand their distress and find ways to personalise care to manage and reduce symptoms of distress. Families recognised the presence or absence of personalised care to their relative which impacted their experience of the inpatient stay.

#### 3.2.1 Subtheme 1: ‘We're a Very Skilled Team’

When staff knew patients well, they believed they were able to anticipate and respond to that individual's need in the moment, using the way they communicated, the ward environment and meaningful activities to prevent the escalation of distress. However, it was difficult for new and agency staff to respond appropriately to individuals in the moment, which could increase distress. The personalised approach was often difficult to verbalise. Staff and managers acknowledged that restrictive techniques, such as medication and physical restraint, were needed at times of crisis, while therapy staff felt there could be a tendency to revert to a medical rather than biopsychosocial model of care, especially when wards were short staffed.

we have very dedicated team … despite of all that behaviour, we’ve managed to keep the calm and collective environment (P011, ward manager)

it’s still on a medical model to be honest with you … we are trying to change that into a biopsychosocial model (P009, physiotherapist)

#### 3.2.2 Subtheme 2: ‘I Feel Like When the Team Works Best, It’s Because They’re Working in a Really Communicative and Person‐Centred Way’

Participants from all groups reported that personalising care required good communication within the team, valuing all expertise regardless of seniority, including the voice of families and the person with dementia. Managers, therapists and medical staff reported that regular multidisciplinary team meetings involving all staff members and family supported this communication, with reports and feedback provided where individuals could not attend. However, therapists and families reported that the ward culture was dependent on the ward manager and/or consultant psychiatrist, and could be hierarchical, focus on biomedical approaches to distress management and did not always value family expertise.

co‐produced formulation and then everybody's really on board with how to manage the difficulty then (P004, music therapist)

So this [discharge planning] had all gone on without me knowing (P012, family member)

#### 3.2.3 Subtheme 3: ‘There's Only so Much Time and Resources We've Got’

All participants reported that having enough staff, time and physical resources was essential to delivering personalised care. This meant not just those required to meet the physical health needs of individuals but staff to prepare and deliver activities. There were significant differences in access to staff and resources between wards.

we were lucky … to have activity workers that were separate to the nursing staff (P049, ward manager)

it gets to the point where often the staff are … bringing stuff [equipment for meaningful activities] in themselves (P048, clinical psychologist)

### 3.3 Theme 3: ‘Music Is a Part of Our Life’

Music used by all was reported by all participants' groups to be an accessible, versatile and helpful tool that could support personhood and the delivery of personalised care, thus helping to assess and manage distress. Participants emphasised the importance of individualising the music, with musical tastes often gathered at admission and described the potential negative effect of the wrong music. Managers and staff reported that music was used in group sessions and spontaneously, but there was rarely training and intentional use of music to reduce distress without input from a music therapist.

#### 3.3.1 Subtheme 1: Music Is Used for ‘Distress Mitigation [and] … to Help People to … Be Engaged in Something’

Most participants reported that music could be used by staff to help prevent distress through lifting mood, regulating arousal, providing stimulation, engagement, and opportunities for expression and connection, continuing up to the end of life. Additionally, professionals reported the use of music, in particular personalised songs, could calm individuals if distress levels were escalating but not yet heightened, potentially preventing the need for more restrictive interventions, such as pro re nata (PRN) medication and one‐to‐one observation. Staff, families and music therapists talked about how shared musical interactions could overcome communication barriers, creating community in a place that is often isolating and where some people are very withdrawn. This supported wellbeing for all, and enabled staff to learn more about the individual and ways they could use music in everyday care.

Every situation it can be applied to, there's no limitations … with music (P047, ward manager)

it does really help to build those relationships and have those conversations that are more social, not just … needs led (P048, clinical psychologist)

#### 3.3.2 Subtheme 2: ‘We Haven't Got the Infrastructure Anyway to Play Music’

However, staffing and equipment impacted the use of music. There was disparity between wards. Access to instruments and technology that could support the use of music, could be limited by funding, Trust policies and poor Wi‐Fi. In some cases, staff were reliant on personal phones and mobile data to access music streaming services. Additionally, all participant groups reported that the use of music was reliant on the musical interests and confidence of individual staff members.

The staff are using their phones (P024, assistant practitioner)

time, space and resources … is really important (P001, music therapist)

#### 3.3.3 Subtheme 3: ‘We [Music Therapists] Do the Specialist Therapy … Advise Other People on More Generic Activities’

Where present, embedding a music therapist in the multidisciplinary team could bring a positive, strengths‐based approach and support the use of music in personalised care. Where they had access to wards, music therapists delivered group and individual therapy sessions, which enhanced the benefits of the musical interactions for people with dementia, families and staff. They could formalise the use of music within an individuals' care plan, train staff, contribute to formulations and assessments of distress and communicate with discharge destinations. While understanding of the role of a music therapist was mixed, observation of sessions was the most reported way to raise awareness as well as time and perseverance from the music therapist.

I've been told that people who would never attend or engage in any other group, they would attend music therapy groups (P001, music therapist)

one to ones are the most beneficial because … the referrals are … people who are withdrawn and get distressed easily (P002, music therapist)

## 4 Discussion

This qualitative study is the first to explore experiences of distress and how this is currently managed in inpatient mental health dementia care, including ways in which music and music therapy are used. Findings integrate the perspectives of people with dementia, families, staff and managers in an area where there is a lack of research to support care delivery. They are corroborated with participants, the MELODIC co‐design group and a Dementia Inpatient Experience Group [2, 18, 19, 20, 21].

Our findings highlight the complexity of care needs and multifaceted distress experienced by people with dementia on these wards, supporting previous literature [1, 2]. The Royal College of Psychiatry and British Psychological Society standards for NHS inpatient mental health dementia wards stress the need for a psychological approach to care, prioritising prevention and timely delivery of treatments to patients [9, 10]. However, this was not consistently experienced by patients, staff or families. Staff and families did view distress as a communication of unmet biopsychosocial need, aligning with descriptions of distress by people with dementia and their families [4, 5, 6, 7]. However, the purpose of the admission, to assess and identify unmet needs and formulate care plans to reduce and manage distress, was not always met due to complexity of need and lack of resource. Admission was sometimes thought to be detrimental to the person's wellbeing while discharge was complex, with difficulties obtaining funding and finding placements that could meet the individual's need.

Staff and families sought to identify and meet unmet needs by personalising care, based on a professional love and respect for the person with dementia. Good communication within the multidisciplinary team and collaboration with family members was required to support this. This reflects person‐centred theories developed by Kitwood who stated that ‘there is only one all‐encompassing need—for love’ [28, 29] (p.92). Music was one way that staff reported being able to tailor care to the individual, especially through playing patient‐preferred music, to help calm distress and prevent the use of restrictive interventions. Music therapists discussed how they could support this through assessing how music could be used most helpfully for an individual and contributing to multidisciplinary care plans. The mechanisms through which music could help meet unmet needs, such as lifting mood, regulating arousal and providing stimulation and connection, support programme theory outlining how and why music therapy reduces distress for people with advanced dementia [19].

However, experience and care for people with dementia and families could vary greatly between wards. Some staff, particularly therapy staff, reported the difficulty of changing a medical understanding of responses to distress which was often determined by the ward managers and consultant psychiatrist. Factors which impacted the staff team's ability to personalise care, thus reinforcing a task‐focussed approach, included a lack of staff to support meaningful activities, insufficient communication with families, inappropriate ward environments and limited physical resources, supporting previous findings [1]. Disparities included the presence of a music therapist who, where present, could support staff to embed tailored music in everyday care. Wards without this resource did not have a systematic approach to music use, instead relying on individual staff without formal training. Our data support previously reported implementation barriers to psychosocial interventions on mental health dementia wards, including lack of staff time and high staff turnover [18]. There are also consistencies with barriers identified in general hospital care for people with dementia, including unsuitable environments, lack of resource, and staff shortages, although lack of knowledge and skill was not reflected in our findings perhaps due to the specialist nature of these mental health wards [30]. As a lack of resource for dementia care was reported in the UK more broadly, including on these NHS specialist wards, any psychosocial interventions, including music therapy, will need to explore cost effective ways to be sustainably implemented within the current resource limitations experienced on these wards.

### 4.1 Limitations

This research was conducted in NHS Trusts so generalisability of findings to private healthcare providers and internationally is not known. There could be recruitment bias, with wards delivering good quality care more likely to participate in the research. Researcher bias could also be a factor as all data were collected by music therapists, who also led the initial analysis. Additionally, a formal sampling frame was not utilised, although researchers sought to include participants representing the wide range of roles involved in providing care on mental health dementia wards. Reflexive thematic analysis was led by N.T., and while this was scrutinised by E.W. and M.H.H. and the co‐design team to ensure trustworthiness and explore potential biases and assumptions, interrater reliability of the coding was not tested. To mitigate this, findings were corroborated with people with personal and professional experience of multiple wards. While many ward staff and managers participated, fewer doctors, family members and people with dementia were represented. In particular, including people with dementia in a semi‐structured interview was not always appropriate due to communication and/or cognitive difficulties. The researchers ensured that the voices of families and people with dementia were represented throughout the results and corroborated with the co‐design group. However, future studies should work alongside experts‐by‐experience to consider creative and accessible ways to include the voices of people with dementia. These could include using song and musical interaction as well as images and visual cues to support communication.

### 4.2 Implications for Practice, Policy and Research

Personalising care, including using personalised music, could minimise the use of restrictive techniques such as medication and restraint. This has healthcare and cost implications which could impact patient, staff and family wellbeing [15]. The need for adequate staffing and resources to enable personalised delivery of care for the person with dementia, with families included as experts in their relative's care, should be reflected in practice and policy. This should include funding for specialist interventionists, access to creative resources to support the use of meaningful activities, and dementia‐friendly ward environments. Evidence‐based best practice guidelines for inpatient mental health dementia wards should be developed, including support needs on admission and discharge. Research should also explore how psychosocial interventions, including music and music therapy, can be effectively implemented in everyday care to reduce distress and training needs for staff. This should include ways in which a music therapist can enable and embed the assessment and delivery of personalised music interventions in everyday care. These wards exist within a large care infrastructure, and improvements in community care for people with dementia could prevent admission and reduce delays in discharge. This would have implications for quality of care and inpatient experience for those who are admitted for specialist support on these wards.

### 4.3 Concluding Statement

Inpatient mental health dementia wards in the NHS provide specialist care for people experiencing complex and multi‐faceted distress, yet are neglected in research, policy and funding. To deliver personalised approaches to managing distress, based on a loving and respectful relationship with the person with dementia and their families, including the use of personalised music, wards need adequate funding, resources and staffing. Future research should support the development of standardised best practice guidelines for psychosocial interventions, including the delivery of music therapy, in these settings.

## Author Contributions

All authors contributed to study design. N.T. and M.H.H. conducted data collection. N.T. and S.N. transcribed the data. N.T., M.H.H. and E.W. conducted the initial analysis, all authors contributed to consolidation and interpretation of findings. N.T. draughted the manuscript. All authors read, edited and approved the final manuscript.

## Conflicts of Interest

The authors declare no conflicts of interest.

## Transparency Declaration

I, lead author, affirm that the manuscript is an honest, accurate, and transparent account of the study being reported; that no important aspects of the study have been omitted; and that any discrepancies from the study as planned have been explained.

## Supporting information

Supporting Information S1

## Data Availability

The data are not publicly available due to their containing information that could compromise the privacy of research participants.

## Endnotes

^1^
Music therapy is a psychosocial intervention, delivered by a registered, accredited therapist, recognised in best practice guidelines as a therapy that could support wellbeing [13].

^2^
NT has a Master of Arts and identifies as female. MHH has a Doctorate and identifies as male.
