# Supplementary material for: Experiences and Management of Distress and the Use of Music, Including Music Therapy, on NHS Inpatient Mental Health Dementia Wards: A Qualitative Study
Source: Int J Geriatr Psychiatry. 2025 May 2;40(5):e70091. doi: 10.1002/gps.70091 (PMC12048746; doi:10.1002/gps.70091)
Supplement: Supplementary file 1 — Supporting Information S1 [file GPS-40-e70091-s001.docx]

Music can be a helpful tool to support people with dementia. Music therapy is an intervention delivered by a music therapist who is registered with a body called the Health and Care Professions Council, but using music activities suggested by the therapist in the individual’s care is everyone’s business.

Music therapists will work alongside the individual, team of professionals and family caregivers supporting the individual to inform their treatment. In a similar way to how physiotherapists work, music therapists provide structured sessions to reach a clear goal which is individualised and responsive to need in the moment. This could be in group or individual sessions depending on the person’s need. They can then advise the individual and those supporting them of ways music can be used in their daily lives to support wellbeing, a bit like how doctors might prescribe a drug. This could be to support aspects of care, such as dressing, washing, walking and eating, and to support the use of person-centred activities throughout the week.

Music therapists work with musical sounds and word. With people with dementia this often includes listening to or singing someone’s favourite music, as well as performing, composing and improvising music together. Music therapy might be quiet and peaceful, for example matching the person’s breathing with musical sounds during times of illness or at the end of life. Using known music can be helpful to bring back memories, express emotions and connect with someone’s social, cultural and spiritual identity. Making new music together can be a way of expressing how someone is feeling in the moment when words might be difficult.
